# Potential Time and Recall Benefits for Adaptive AI‐Based Breast Cancer MRI Screening

**DOI:** 10.1002/jmri.70034

**Published:** 2025-07-07

**Authors:** Luuk Balkenende, Jonatan Ferm, Vivien van Veldhuizen, Joren Brunekreef, Jonas Teuwen, Ritse M. Mann

**Affiliations:** ^1^ Department of Radiology Netherlands Cancer Institute (NKI) Amsterdam the Netherlands; ^2^ Department of Medical Imaging Radboud University Medical Center Nijmegen the Netherlands; ^3^ Department of Radiation Oncology Netherlands Cancer Institute (NKI) Amsterdam the Netherlands; ^4^ Faculty of Science University of Amsterdam Amsterdam the Netherlands

**Keywords:** artificial intelligence, breast cancer screening, expected protocol duration, recall rate, screening protocol, theoretical limits

## Abstract

**Background:**

Abbreviated breast MRI protocols are advocated for breast screening as they limit acquisition duration and increase resource availability. However, radiologists' specificity may be slightly lowered when only such short protocols are evaluated. An adaptive approach, where a full protocol is performed only when abnormalities are detected by artificial intelligence (AI)‐based models in the abbreviated protocol, might improve and speed up MRI screening. This study explores the potential benefits of such an approach.

**Purpose:**

To assess the potential impact of adaptive breast MRI scanning based on AI detection of malignancies.

**Study Type:**

Mathematical model.

**Field Strength/Sequence:**

Breast cancer screening protocols.

**Assessment:**

Theoretical upper and lower limits on expected protocol duration and recall rate were determined for the adaptive approach, and the influence of the AI model and radiologists' performance metrics on these limits was assessed, under the assumption that any finding on the abbreviated protocol would, in an ideal follow‐up scenario, prompt a second MRI with the full protocol.

**Statistical Tests:**

Estimated most likely scenario.

**Results:**

Theoretical limits for the proposed adaptive AI‐based MRI breast cancer screening showed that the recall rates of the abbreviated and full screening protocols always constrained the recall rate. These abbreviated and full protocols did not fully constrain the expected protocol duration, and an adaptive protocol's expected duration could thus be shorter than the abbreviated protocol duration. Specificity, either from AI models or radiologists, has the largest effect on the theoretical limits. In the most likely scenario, the adaptive protocol achieved an expected protocol duration reduction of ~47%–60% compared with the full protocol.

**Data Conclusion:**

The proposed adaptive approach may offer a reduction in expected protocol duration compared with the use of the full protocol alone, and a lower recall rate relative to an abbreviated‐only approach could be achieved. Optimal performance was observed when AI models emulated radiologists' decision‐making behavior, rather than focusing solely on near‐perfect malignancy detection.

**Evidence Level:**

Not applicable.

**Technical Efficacy:**

Stage 6.

**Summary**

- Plain language summary
  ○ This study looked at a new way to improve breast cancer screening using magnetic resonance imaging (MRI).
  ○ Full MRI scans are accurate but take longer, while shorter scans save time but may miss some details.
  ○ We tested an adaptive approach using artificial intelligence (AI).
  ○ In this method, all patients first get a short MRI scan.
  ○ Only if the AI detects something unusual, a full scan is done.
  ○ The results showed this approach could save time and reduce unnecessary follow‐up imaging without lowering quality.
  ○ These findings suggest AI can help make breast cancer screening faster and more efficient.

## 1 Introduction

Breast cancer is a leading cause of mortality among women worldwide, with over 660,000 deaths globally in 2022 [1]. To reduce breast cancer mortality, screening programs have been established to enable early detection, making treatment more effective and improving survival rates compared with cancers diagnosed in late stages [2]. Dynamic contrast‐enhanced breast magnetic resonance imaging (DCE‐MRI) offers higher sensitivity and fewer interval cancers compared with mammography, especially for women at high risk of breast cancer [3, 4]. Despite its advantages, the use of breast DCE‐MRI is limited due to relatively high costs, making mammography the primary tool in most screening programs [5].

An abbreviated breast MRI protocol has been proposed to reduce costs and improve accessibility [6]. This protocol includes pre‐contrast and first post‐contrast T1‐weighted images, and may also incorporate interleaved ultra‐fast inflow imaging. By reducing the number of sequences, the total screening time is shortened from ~13 min to about 4 min [7]. While this abbreviated protocol is expected to maintain sensitivity, the absence of additional sequences such as multiple post‐contrast T1‐weighted, T2‐weighted, and diffusion‐weighted sequences may reduce specificity and omit valuable prognostic and predictive information [8, 9].

An adaptive screening approach, where a full protocol is performed only if abnormalities are detected in an initially abbreviated protocol, could combine the efficiency of the abbreviated protocol with the higher specificity of the full protocol. However, making rapid go/no‐go decisions—whether to proceed with scanning with a full protocol—is helpful to avoid missing contrast outflow timing or unnecessarily prolonging scanning when no abnormalities are present. Artificial intelligence (AI), particularly deep learning (DL), could assist in quickly and accurately detecting abnormalities, enabling efficient decision‐making [10, 11].

Specifically, DL models have shown promise in breast cancer detection, primarily in mammography [12]. Although some MRI‐based DL detection models exist [13, 14], their clinical application for detecting small tumors (typically < 20 mm) in screening MRI remains limited [15]. Before implementing dedicated DL models for the proposed adaptive breast cancer MRI screening, it is crucial to evaluate their potential impact. Establishing theoretical limits for recall rate (RR) and expected protocol duration (EPD) can help assess the feasibility of adaptive screening. Additionally, understanding how DL model performance influences these limits can guide optimization efforts.

Therefore, this study aimed to determine the theoretical limits of EPD and RR for an adaptive AI‐based breast MRI screening approach, and to analyze the impact of DL model and radiologist performance on these limits.

## 2 Materials and Methods

No real‐world data from individual people was required for this study. Therefore, no institutional review board or ethical review was required. Hence, the requirement for written informed consent was voided.

### 2.1 Overview

This study evaluated the potential benefits of an AI‐based adaptive breast screening protocol compared with either an abbreviated protocol or a full protocol alone. In detail, the terms abbreviated‐only and full‐only are occasionally used in this manuscript to explicitly refer to these standalone protocol types.

The abbreviated protocol consisted of T1‐weighted pre‐contrast and first post‐contrast scans. In contrast, the full protocol included these sequences along with three additional post‐contrast T1‐weighted scans, one T2‐weighted scan, and one diffusion‐weighted imaging (DWI) sequence to provide further prognostic information. The adaptive protocol combined elements of both approaches by determining whether to proceed with additional imaging to obtain the remaining sequences of the full protocol—immediately after completing the abbreviated protocol.

The assessment focused on two key metrics: RR and EPD. The RR represented the percentage of people recalled for imaging with the full protocol after initially undergoing only the abbreviated protocol. Specifically, people were recalled when radiologists identified a suspicious lesion on the abbreviated protocol based on Breast Imaging Reporting and Data System (BI‐RADS) criteria [16], as would occur in an ideal follow‐up scenario. The EPD was defined as the average time required for one exam, including the time spent scanning recalled people.

While the RR and EPD can be directly calculated for the abbreviated‐only and full‐only protocols, they cannot be precisely determined for the adaptive protocol. Instead, upper and lower limits for these metrics were determined by analyzing best‐case and worst‐case scenarios.

### 2.2 Best‐Case and Worst‐Case Scenarios

#### 2.2.1 Best‐Case Scenario

In the best‐case scenario for the adaptive protocol, full protocol imaging was performed only for people in whom radiologists suspected malignant findings based on the abbreviated protocol. Patients without suspicious findings completed only the abbreviated protocol, while those with suspicious findings underwent immediate full protocol imaging. This scenario achieved the lowest possible EPD, as the full protocol was applied only in cases with suspicious findings, as identified by radiologists. It also minimized the RR because additional imaging was promptly performed for patients in whom radiologists deemed the full protocol necessary.

This approach implied that a DL detection model used for protocol‐type selection in the adaptive protocol must flag the same patients as radiologists would for full protocol scanning based on the abbreviated protocol. Achieving this required the DL model's sensitivity and specificity to be identical to those of the radiologists—a condition rarely met in practice. Thus, the best‐case scenario was characterized by the maximum possible overlap between patients selected by the DL model and those identified by radiologists, given the performance metrics of both the DL model and radiologists.

#### 2.2.2 Worst‐Case Scenario

In the worst‐case scenario for the adaptive protocol, patients with suspicious findings were initially imaged using only the abbreviated protocol, while patients without suspicious findings underwent the full protocol. This scenario resulted in the highest possible RR, as all people with suspicious findings by radiologists must be recalled for further imaging. It also maximized the EPD since all people underwent full protocol imaging in the end, with people flagged with suspicious findings by radiologists completing both the abbreviated and full protocols.

Unlike the best‐case scenario, the worst‐case scenario was characterized by the minimum possible overlap between people flagged by the DL model and those identified by radiologists.

### 2.3 Limits Calculation

The corresponding maximum and minimum overlaps between the DL model and radiologists were converted into RR and EPD values.

#### 2.3.1 Recall Rate (RR)

The RR range for the adaptive protocol was calculated using Equation (1).

(1) $${\mathrm{RR}}_{\text{adaptive}}=\frac{\left(tp_{\text{abbr}}+fp_{\text{abbr}}\right)-\text{overlap}}{N}$$

where $tp_{\text{abbr}},fp_{\text{abbr}}$ represented the true and false positives of the radiologists on the abbreviated protocol, respectively; overlap referred to the overlap between the true and false positives of the DL detection model and radiologists on the abbreviated protocol, and could range between the minimum and maximum possible overlap; and N was the total number of people.

The RR was defined as the proportion of patients who needed the full protocol but did not receive it immediately, relative to the total number of patients. Individuals requiring full protocol imaging were those recalled by radiologists based on the abbreviated protocol (i.e., both true positives and false positives). The number of patients who had already undergone full protocol imaging was determined by the overlap. Greater overlap corresponded to a lower RR, while smaller overlap resulted in a higher RR.

We also determined the RR for the full‐only and abbreviated‐only protocols. When the full protocol was scanned in all people, the RR was 0, leaving no additional MRI scans of these patients to be acquired. When all people were scanned with the abbreviated protocol, the RR was given by the proportion of people in whom the radiologists flagged a finding (both true positives and false positives) on the abbreviated protocol.

#### 2.3.2 Expected Protocol Duration (EPD)

The EPD range was calculated using Equation (2).

(2) $$\begin{array}{c} {\mathrm{EPD}}_{\text{adaptive}}=\frac{t_{\text{full}}\left(tp_{\mathrm{ai}}+fp_{\mathrm{ai}}+\mathrm{RR}\right)+t_{\text{abbr}}\left(tn_{\mathrm{ai}}+fn_{\mathrm{ai}}\right)}{N} \end{array}$$

where $tp_{\mathrm{ai}},fp_{\mathrm{ai}},tn_{\mathrm{ai}},fn_{\mathrm{ai}}$ were the true positives, false positives, true negatives, and false negatives, respectively, of the DL model; $t_{\text{full}},t_{\text{abbr}}$ were the acquisition durations of the full and abbreviated protocols, respectively; RR referred to the recall rate of the adaptive protocol, as given by Equation (1); and N was the total number of people.

First, the total protocol duration was calculated by accounting for all patients who underwent imaging. Some people were imaged using only the abbreviated protocol, while others underwent the full protocol. Additionally, when radiologists identified findings missed by the DL model, those individuals—initially scanned with only the abbreviated protocol—were recalled for a full protocol scan. In such cases, they were effectively scanned twice with the abbreviated protocol. Finally, this total protocol duration was divided by the total number of people to arrive at the EPD.

We also determined the EPD for the full‐only and abbreviated‐only protocols. In the case of the full protocol, this was simply the full protocol duration. For the abbreviated protocol, the EPD was obtained by averaging the total abbreviated scan time and the time added due to recalls for full protocol imaging, over all individuals.

### 2.4 Statistical Analysis

This study is theoretical in nature and does not involve statistical hypothesis testing or data‐driven inference. Instead, the analysis focuses on the derivation and evaluation of theoretical RR and EPD bounds for different MRI protocol strategies under varying DL model performance assumptions.

To provide a practical reference for interpreting the results, we included an estimated performance range for the adaptive protocol based on existing literature. Specifically, we compiled reported accuracy values from studies reviewed by Adam et al. [15], yielding a mean accuracy of 83.4% with a 95% confidence interval of ~75%–91% (*n* = 14). This interval is used to indicate the region within which an AI model trained to emulate radiologist decision‐making would most likely perform. This allows for a more grounded interpretation of where the adaptive protocol might operate in practice, relative to its theoretical best‐ and worst‐case performance.

The parameters used in these calculations—including scanning duration, radiologists' performance on the abbreviated and full protocols, and malignancy rate—are listed in Table 1. As realistic values for the sensitivity and specificity of the DL model were not yet known, these were kept as free parameters that could be varied in our calculations.

**TABLE 1:** Parameters for sensitivity, specificity, scanning duration, and malignancy rate used in limit determination.

Full protocol	Sensitivity	92% [8]
	Specificity	95% [8]
	Scanning duration	776 s [7]
Abbreviated protocol	Sensitivity	90% [8]
	Specificity	92% [8]
	Scanning duration	262 s [7]
Malignancy rate in screening		1.46% [17]

The literature reported a wide range of values for radiologists' sensitivity and specificity on the abbreviated protocol, as well as varying durations for abbreviated and full protocols [6, 9, 18, 19, 20, 21, 22, 23]. To account for this variability, we performed additional experiments to investigate the effect of changes in these parameters. In this second set of experiments, the DL detection model's sensitivity and specificity were fixed at 80%.

All calculations and figure generation were performed using Python, and the analysis code is publicly available on Github at https://github.com/luukbalkenende/Adaptive‐MRI‐bounds.

## 3 Results

### 3.1 Effect of AI Specificity and Sensitivity

**FIGURE 1:**
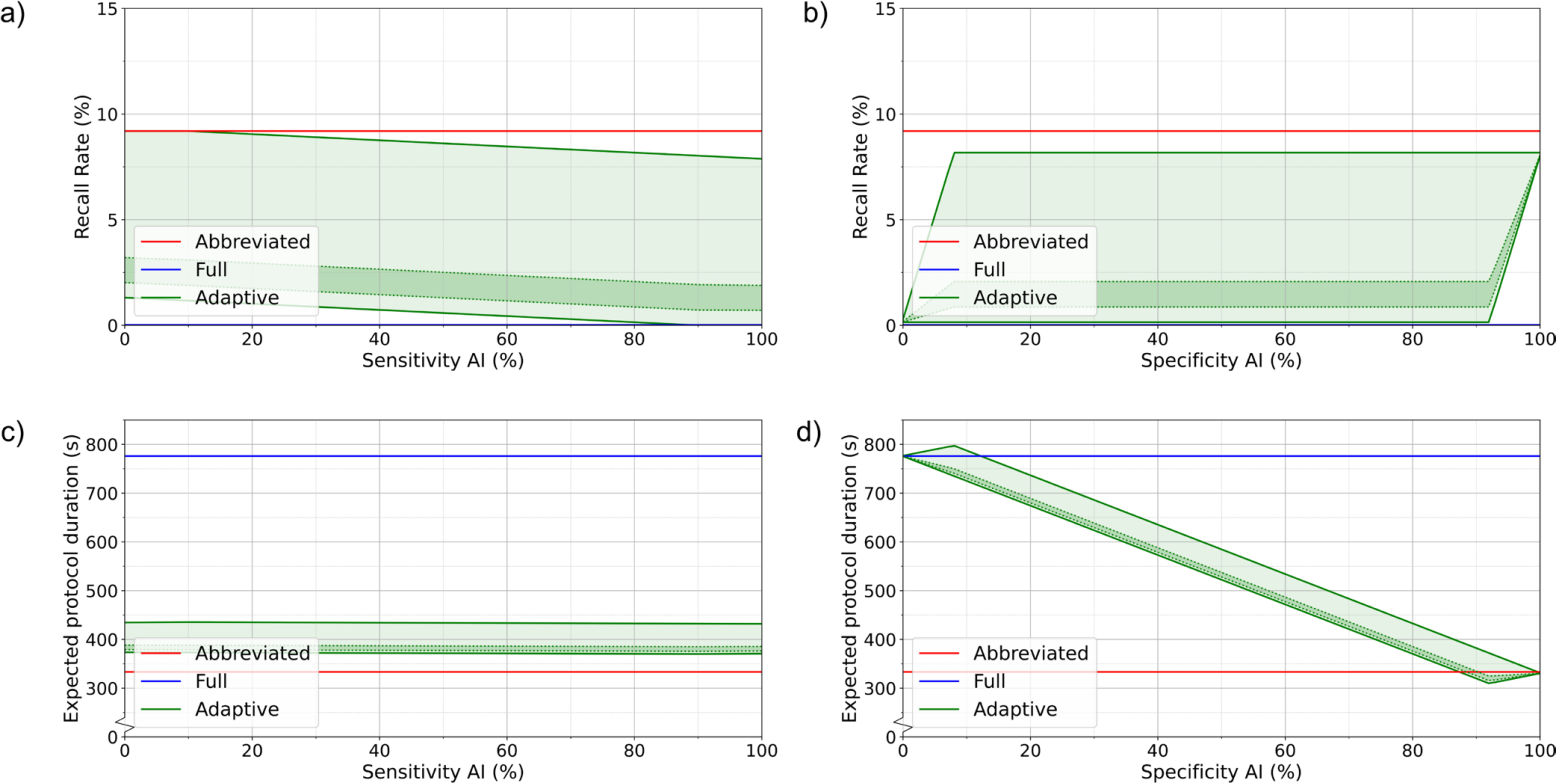
Effect of AI model sensitivity and specificity on recall rate (RR) and expected protocol duration (EPD). Red and blue lines represent the cases of only abbreviated and full protocols, respectively. Green lines represent the upper and lower theoretical limits of the adaptive protocol, with the green area indicating the full possible range. The darker green band between the dotted lines highlights the estimated most likely range. (a) Effect of AI sensitivity on RR, (b) effect of AI specificity on RR, (c) effect of AI sensitivity on EPD, and (d) effect of AI specificity on EPD.

The upper and lower limits of the RR for the proposed adaptive protocol were bounded by the constant RR values of the abbreviated and full protocols, respectively, as seen in Figure 1a,b. This behavior was expected since the adaptive protocol is a hybrid approach between these two protocols.

In Figure 1a, AI sensitivity inversely affected RR limits; increasing AI sensitivity reduced both limits linearly. When AI sensitivity matched radiologists' sensitivity on the abbreviated protocol (90%), the lower limit reached zero (the same as the full protocol) and could not decrease further. Similarly, when AI sensitivity is lower than 10% (equal to 100% minus radiologists' sensitivity), the upper limit was equal to the RR of the abbreviated protocol.

Figure 1b shows that AI specificity influenced RR limits in two regimes. Between 0% and 8% specificity (100% minus radiologists' abbreviated protocol specificity), the upper limit rose linearly to its maximum. From 92% to 100%, the lower limit increased similarly. Notably, an AI specificity of 100% did not achieve the lowest possible RR in the adaptive setting.

The darker region between the dotted green lines indicated the most likely overlap in performance between radiologists and the AI model. Using these bounds, the RR was estimated to fall most likely between 1% and 2% for the adaptive protocol.

Unlike RR, the EPD limits for the adaptive protocol were not confined by the durations of the abbreviated and full protocols (Figure 1c,d). The adaptive EPD could be shorter than the abbreviated protocol since some individuals underwent the full protocol upfront, avoiding recall. Conversely, it could exceed the EPD of the full protocol if initially abbreviated scanned cases required recall for the full protocol.

The AI specificity had a greater impact on EPD than sensitivity (Figure 1d), with both limits decreasing linearly as specificity increased. In the outer regimes, the upper limit rose between 0% and 8%, while the lower limit increased from 92% to 100%, reflecting radiologists' abbreviated protocol specificity (92%) and its influence on false positives.

The AI sensitivity minimally affected EPD (Figure 1c) but followed the same pattern as specificity—higher sensitivity reduced EPD. In the outer regimes, the upper limit increased between 0% and 10%, and the lower limit raised from 90% to 100%, driven by radiologists' 90% sensitivity on the abbreviated protocol.

When examining the darker region—representing the most likely overlap in performance between radiologists and AI—the EPD for the adaptive protocol was estimated to lie most likely between 310 and 410 s. This corresponds to an EPD reduction of ~47%–60% compared with the full protocol.

### 3.2 Effect of Radiologists' Sensitivity and Specificity of Abbreviated Protocol

**FIGURE 2:**
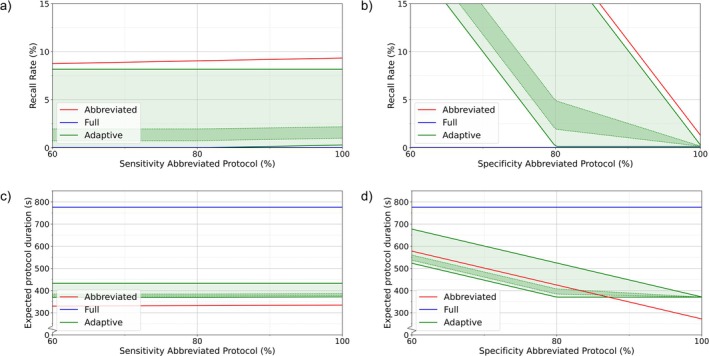
Effect of radiologists' sensitivity and specificity on abbreviated protocol on recall rate (RR) and expected protocol duration (EPD). Red and blue lines represent the cases of only abbreviated and full protocols, respectively. Green lines represent the upper and lower theoretical limits of the adaptive protocol, with the green area indicating the full possible range. The darker green band between the dotted lines highlights the estimated most likely range. (a) Effect of sensitivity on RR, (b) effect of specificity on RR, (c) effect of sensitivity on EPD, and (d) effect of specificity on EPD.

The RR of the abbreviated protocol increased with higher sensitivity (Figure 2a) since more individuals were correctly recalled for a full protocol. The adaptive protocol's RR limits remained constant until radiologists' sensitivity surpassed AI sensitivity (80%), causing the lower limit to rise as radiologists recalled cases missed by AI.

Radiologists' specificity had the opposite effect—higher specificity reduced RR by minimizing incorrect recalls (Figure 2b). The upper limit of the adaptive protocol remained bounded by the abbreviated protocol, as was already observed in Figure 1a,b. However, when radiologists' specificity dropped below AI specificity (80%), the lower limit of the adaptive protocol began to rise. This occurred because radiologists recalled more individuals than those already selected for full imaging by the adaptive protocol.

Radiologists' specificity had a greater impact on the EPD than their sensitivity due to the low prevalence of breast cancer in screening populations (Figure 2c,d). Consequently, changes in specificity affected more individuals than equivalent changes in sensitivity. Again, the EPD limits for the adaptive protocol were not bounded by the abbreviated and full protocols, although they followed a similar trend as the abbreviated protocol and decreased linearly with increasing specificity. When radiologists' specificity surpassed AI specificity, the lower limit became a constant. Here, radiologists would not recall more people in the adaptive setting, resulting in a constant EPD.

### 3.3 Effect of Protocol Duration

**FIGURE 3:**
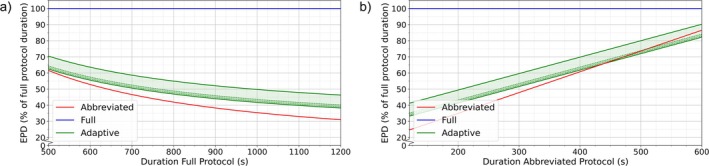
Effect of protocol duration on expected protocol duration (EPD). Red and blue lines represent the cases of only abbreviated and full protocols, respectively. Green lines represent the upper and lower theoretical limits of the adaptive protocol, with the green area indicating the full possible range. The darker green band between the dotted lines highlights the estimated most likely range. (a) Effect of full protocol duration, (b) effect of abbreviated protocol duration. *Y*‐axes are given in the percentage of full protocol duration.

Figure 3a,b, show the impact of full and abbreviated protocol durations on EPD. A longer full protocol duration favored the abbreviated‐only protocol setting compared with the adaptive protocol. Conversely, a longer abbreviated protocol duration favored the adaptive setting over the abbreviated‐only protocol. This occurred because the ratio of full to abbreviated protocol scans was smaller in the abbreviated protocol setting, and changes in protocol duration further amplified this difference between settings.

## 4 Discussion

This study showed that an AI‐based adaptive breast MRI protocol could reduce RR while maintaining or improving time efficiency compared with using only an abbreviated protocol. The theoretical limits on the RR for the proposed adaptive protocol were bounded by the RR of the abbreviated and full protocol settings, which means that the RR for the adaptive setting would always be lower than that for the abbreviated setting. The EPD, however, was not bound by the durations of the abbreviated and full protocol settings. Theoretically, the adaptive protocol duration could thus be shorter than the EPD of the abbreviated setting. Specificity (both radiologists' and AI's) had a greater impact on EPD than sensitivity.

Most AI research in breast cancer detection focused on maximizing sensitivity and specificity, with AI in mammography beginning to outperform radiologists, enabling standalone screening [12, 24, 25]. However, this study showed that employing a near‐perfect AI model as a go/no‐go decision‐maker at the scanner may not achieve the optimal outcome for the proposed adaptive protocol. More specifically, the best results occurred when AI performance closely matched radiologists' performance. When AI specificity surpassed radiologists', the lower limit on RR increased, subsequently increasing the lower limit of the EPD, moving away from the optimal point.

The wide range of RR and EPD limits suggests that even with AI performance on par with radiologists, the theoretical optimum may not be reached. Achieving the lower limits in practice requires the AI model to select the same individuals for full protocol scanning as radiologists would. This highlights the importance of developing AI models that emulate radiologists' decision‐making. Instead of relying solely on malignancy scores, AI models should be trained using indicators like the BI‐RADS score [16], which reflects the radiologists' judgment for additional scans. Training AI on this score would better align its decisions with those of radiologists, bringing the adaptive protocol closer to the theoretical optimal performance.

The simplicity of the theoretical model allows for clear visualization and understanding of how varying radiologists' performance metrics could influence outcomes. Even when radiologist performance dropped significantly, the theoretical limits maintained consistent trends. This emphasizes again the importance of aligning AI behavior to that of radiologists, regardless of radiologists performance.

Notably, greater potential gains in RR and EPD can be achieved in settings where radiologists' performance was lower. However, these gains are accompanied by increased risk, as the adaptive protocol may underperform compared with the abbreviated protocol if AI fails to adequately emulate radiologists, leading to an increased RR or unnecessary full‐protocol scans. Conversely, in breast cancer screening in clinical practice, where radiologists typically exhibit high performance, the potential gains could be smaller, but the associated risks (AI‐induced errors) should also be reduced. This indicates that even when AI does not perfectly emulate radiologists, the adaptive protocol could still yield meaningful improvements in EPD. Future work should explore the extent to which AI will deviate from radiologists' decisions.

To advance AI‐based adaptive breast cancer screening with MRI, developing AI algorithms that emulate radiologists' decision‐making is crucial to reduce EPD while maintaining low RR. However, a more comprehensive evaluation of this adaptive approach is necessary. Further research should not only evaluate RR and EPD compared with abbreviated and full MRI protocols, but also to mammography, the standard imaging modality [26]. Additional factors like patient comfort, given mammography's associated discomfort, should also be considered for a fair comparison [26, 27]. The adoption of the presented approach may face fewer trust‐related barriers compared with other AI applications in healthcare, as radiologists retain full control. This method could enhance efficiency without compromising screening outcomes, reducing concerns about AI reliance [28, 29]. However, further implementation studies could confirm these assumptions.

### 4.1 Limitations

We assumed that all suspicious findings from abbreviated protocols (both false and true positives) require full protocol scans. This simplification does not fully reflect clinical practice, where alternative follow‐up strategies, such as other imaging modalities or biopsies, are often used instead of complete MRI. In real‐world implementation, this would likely result in a lower RR for the adaptive protocol, thereby narrowing the gap in RR between the full and adaptive protocol. As a result, one of the main drawbacks of the adaptive protocol—its higher RR—would be mitigated, improving its clinical feasibility. Additionally, it is often accepted that not all MRI sequences are available when a malignancy is detected through screening. This implies that the actual expected scanning time of an abbreviated protocol will be shorter than estimated in our study due to lower recall rates (to be scanned with the full protocol), potentially at the cost of a reduction in available information. Another limitation involves false negatives between abbreviated and full protocols. Although radiologists' sensitivity is assumed to be similar in both, the specific false negatives may differ. This variability suggests that a go/no‐go decision of AI in the adaptive setting could influence radiologists' sensitivity. Consequently, the definition of the best‐case scenario could shift from prioritizing specificity and time reduction to maximizing sensitivity. In this study, we have limited the scope of the best‐case scenario to the former. Finally, the study's theoretical design inherently limits its findings. While our modeling provides valuable insights, clinical implementation—considering the complexities of patient management, decision‐making, clinical workflow, and costs—may yield different outcomes.

## 5 Conclusion

This study assessed the theoretical limits on EPD and RR for adaptive AI‐based MRI breast cancer screening. The results showed that this adaptive approach could reduce EPD compared with a full‐only protocol setting and RR compared with an abbreviated‐only setting. Optimal performance can be achieved when the AI detection model emulates radiologists' decision‐making behavior.
